# SREBP-1c as a molecular bridge between lipogenesis and cell cycle progression of clear cell renal carcinoma

**DOI:** 10.1042/BSR20171270

**Published:** 2017-12-15

**Authors:** Gautam Sethi, Muthu K. Shanmugam, Alan Prem Kumar

**Affiliations:** 1 Department for Management of Science and Technology Development, Ton Duc Thang University, Ho Chi Minh City, Vietnam; 2 Faculty of Pharmacy, Ton Duc Thang University, Ho Chi Minh City, Vietnam; 3 Department of Pharmacology, Yong Loo Lin School of Medicine, National University of Singapore, Singapore 117600, Singapore; 4 Cancer Science Institute of Singapore, National University of Singaporee; 5 Medical Science Cluster, Yong Loo Lin School of Medicine, National University of Singaporea; 6 National University Cancer Institute, National University Health System, Singapore

**Keywords:** lipogenesis, renal cell carcinoma, SREBP-1c

## Abstract

Sterol regulatory element binding protein 1c (SREBP-1c) promotes lipogenesis and tumor growth in various cancers. It is well known that clear cell renal cell carcinoma (ccRCC), a major subtype of the kidney cancers, exhibits elevated lipid accumulation. However, it has not been fully understood how lipid metabolism might be associated with cell cycle regulation in ccRCC. In a recent issue, Lee et al. (Molecular and Cellular Biology (2017) pii: MCB.00265-17) demonstrate that SREBP-1c is up-regulated in ccRCC by ring finger protein 20 (RNF20) down-regulation, leading to aberrant lipid storage and pituitary tumor transforming gene 1 (*PTTG1*)-dependent cell cycle progression. These findings suggest that SREBP-1c serves as a molecular bridge between lipid metabolism and cell cycle control in ccRCC tumorigenesis.

## Overview: sterol regulatory element binding protein 1c and tumor growth

Metabolic reprogramming is one of the hallmarks in most cancer cells. Tumor cells exhibit enhanced aerobic glycolysis and elevated lipogenesis to supply energy and building blocks, which are required for excessive cell growth [1]. Sterol regulatory element binding proteins (SREBPs) are key transcription factors that regulate lipid metabolism and energy storage through the synthesis and uptake of fatty acids, triglycerides, and cholesterol. Mammals have three SREBP isoforms: SREBP-1a, SREBP-1c, and SREBP-2. SREBP-1 primarily controls the lipogenic pathway, whereas SREBP-2 is specific for cholesterol biosynthesis [2]. Previously, it has been reported that SREBPs are associated with aberrant lipid metabolism required for tumor growth [3]. For instance, it has been shown that the Akt/mTOR pathway stimulates SREBP-1c-dependent lipogenesis and SREBP-1c is required for cell survival and tumorigenesis in glioblastoma and breast cancers [4]. Although clear cell renal cell carcinoma (ccRCC), a major subtype of the kidney cancer, shows ectopic lipid accumulation [5], the underlying mechanisms of the relationship between aberrant lipid metabolism and tumorigenesis in ccRCC are poorly understood. It has been reported that ring finger protein 20 (RNF20), a RING-finger containing E3 ubiquitin ligase, acts as a tumor suppressor by modulating histone monoubiquitination and epigenetic gene expression [6]. Remarkably, Lee et al. [7] demonstrate that RNF20 specifically stimulates polyubiquitination and degradation of SREBP-1c, thereby negatively regulating hepatic lipogenesis upon PKA activation. The present study provides a clue to understand how SREBP-1c is down-regulated by fasting signals to prevent excess lipogenesis. However, RNF20 had not been previously implicated in lipid metabolism-mediated tumor-suppressive function.

Interestingly, in a recent issue of Molecular and Cellular Biology, Lee et al. [8] provided a new clue between lipid metabolism and cell cycle regulation in tumor growth. They now suggest that SREBP-1c could function as a molecular linker between lipogenesis and cell cycle, particularly in ccRCC. In ccRCC patients, RNF20 down-regulation is a marker of poor prognosis. RNF20 inhibits lipogenesis and cell proliferation by suppressing SREBP-1c in ccRCC. Intriguingly, RNF20 represses cell cycle progression by modulating pituitary tumor transforming gene 1 (*PTTG1*) as a novel SREBP-1c target gene. Thus, the authors have clearly demonstrated that RNF20 acts as a metabolic tumor suppressor by inhibiting SREBP-1c-mediated lipogenesis and cell cycle progression in ccRCC.

## New insights into SREBP-1c in kidney cancers

Histologically, ccRCC is characterized by the clear cell phenotype due to lipid accumulation, indicating that metabolic perturbations are a defining feature of this tumor. To date, it has been established that the primary etiology of ccRCC is resulted from inactivation of von Hippel–Lindau (*VHL*) tumor suppressor gene and the consequent activation of hypoxia-inducible factor (HIF) [5]. Accordingly, a recent study suggests that HIF-2α promotes lipid storage, endoplasmic reticulum (ER) homeostasis, and cell viability in ccRCC via up-regulation of the lipid droplet protein PLIN2 [9]. Although this study demonstrates the HIF-2α/PLIN2 axis maintains lipid droplets to protect against ER stress in ccRCC, the underlying mechanisms that are involved in ectopic lipid accumulation and cell cycle regulation in ccRCC have not been fully elucidated. In recent work, Lee et al. [8] have investigated whether SREBP-1c and lipogenesis are required in ccRCC tumor growth. SREBP-1c overexpression promotes lipogenesis and proliferation, whereas genetic and pharmacological inhibition of SREBP-1c abrogates these effects in ccRCC cells. Furthermore, The Cancer Genome Atlas (TCGA) reveals that mRNA levels of SREBP-1c and lipogenic genes are up-regulated in ccRCC patients, which is accompanied with advanced tumor stages and poor survival [10]. Mechanistically, it has been found that RNF20 inhibits lipogenesis and cell cycle progression via SREBP-1c in both ccRCC cells and xenograft mouse models. These findings evidently indicate that RNF20 suppresses ccRCC tumorigenesis by inhibiting the SREBP-1c pathway.

Spontaneous deletion of *VHL* is the most common cause of hereditary and sporadic forms of ccRCC [5]. Apart from *VHL* loss, ccRCC patients exhibit remarkable genetic heterogeneity and frequent mutations in various metabolic genes such as *MET, FLCN*, and *TSC1/2* [11]. Given that metabolic reprogramming is tightly associated with ccRCC, this type of tumor is often called a metabolic disease [12]. Moreover, it has been reported that HIF-2α antagonists might be beneficial against ccRCC [13,14]. However, there are ccRCC tumors that are resistant to HIF-2α antagonists in both *VHL* wild-type (SLR21) ccRCC cell lines and patient-derived xenograft tumors with low level of HIF-2α [13,14]. In order to overcome the limited effects of HIF-2α antagonists, alternative therapeutic targets against ccRCC should be elucidated. Lee et al. [8] have identified a novel pathway of SREBP-1c-dependent ccRCC tumor, independent of *VHL* mutation. First, they show that low level of RNF20 is significantly associated with poor prognosis in ccRCC patients, regardless of *VHL* mutation status. Second, RNF20 represses SREBP-1c expression and cell growth in both *VHL* wild-type (ACHN) and *VHL*-deficient (A498) ccRCC cell lines. Last, the SREBP inhibitor betulin suppresses ccRCC proliferation by down-regulating SREBP-1c and lipogenesis with or without *VHL* mutation. Therefore, they elucidate a novel pathway involved in a *VHL*-independent ccRCC tumorigenesis.

Previous studies have shown that SREBP-1c appears to be associated with cell cycle regulation in several aspects. For example, SREBP-1c stimulates expression of key genes involved in cell cycle control [15]. In addition, cell cycle regulated kinases including Cdk1 and Plk1 promote phosphorylation-dependent activation of SREBP-1c during mitosis [16,17]. Moreover, SREBP-regulated miRNAs such as *miR-33* and *miR-182* co-operate to regulate cell proliferation and cell cycle [18,19]. In the recent study, Lee et al. [8] have found that SREBP-1c promotes cell cycle progression by enhancing expression of *PTTG1*, a novel target gene of SREBP-1c. PTTG1, also known as securin, not only prevents premature chromosome separation by inhibiting separase activity, but also promotes cell cycle dysregulation by regulating cell cycle genes [20]. The authors clearly demonstrate that SREBP-1c potently stimulates the expression of several cell cycle genes such as *PCNA* and *cyclin A* in a PTTG1-dependent manner, potentiating cell proliferation in ccRCC. Thus, SREBP-1c–PTTG1 axis provides new insights that SREBP-1c can directly regulate cell cycle in addition to controlling lipid metabolism through its well-known lipogenic targets.

Lipid availability is crucial for cell viability and cell cycle regulation. For instance, unsaturated fatty acids increase cyclin D1 expression and cell proliferation by activating β-catenin in ccRCC [21]. In addition, the inhibitor of lipogenic enzyme SCD1 suppresses tumor growth and invasiveness of ccRCC [22]. Lee et al. also addressed the question whether lipogenesis is required for induction of PTTG1 and cell cycle genes [8]. They found that the expression of PTTG1 and cell cycle genes is not affected by pharmacological inhibition of lipogenesis using the ACC inhibitor TOFA or the FASN inhibitor C75 or by siRNA-mediated suppression of FASN. These results suggest that SREBP-1c separately regulates lipogenesis and PTTG1-mediated cell cycle progression. Taken together, the present work proposes that SREBP-1c serves as a molecular bridge between lipid metabolism and cell cycle regulation by modulating different pathways, which eventually coalesce to drive ccRCC tumorigenesis. Further insight into connection points between the lipid metabolism and cell cycle might pave the way for the development of effective therapies that target metabolic vulnerabilities of ccRCC.

**Figure 1:**
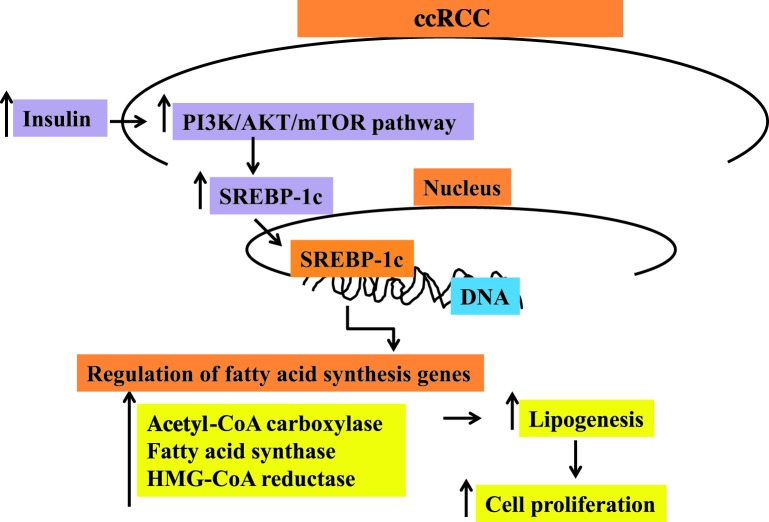
Regulation of SREBP-1c in ccRCC

Another SREBP isoform, SREBP-2 plays a key role in tumor transformation and invasion through mevalonate pathway [23]. As described earlier, ccRCC is characterized by the accumulation of neutral lipids such as triglycerides and cholesterol esters. Previous studies have shown that the activity of esterification of cholesterol is significantly higher in ccRCC than in biosynthesis and uptake of cholesterol [24,25]. In accordance with these, Lee et al. [8] observed that the expression of SREBP-2 and cholesterol metabolism genes such as HMG-CoA reductase and LDL receptor appear to be decreased in ccRCC patients. While further studies on the effect of SREBP-2 on ccRCC tumorigenesis are needed, it is plausible to speculate that SREBP-1c will play more oncogenic roles in ccRCC. Figure 1 briefly summarizes the various signal transduction pathways involved in the regulation of SREBP-1c in ccRCC.

## Future directions

Many studies have reported that SREBP-1c is associated with cell cycle regulation. In recent paper, Lee et al. have identified a novel pathway by which SREBP-1c directly regulates the cell cycle through PTTG1 [8]. Moreover, they have proposed that SREBP-1c seems to regulate lipid metabolism and cell cycle pathways in a separate manner. Although Lee et al. revealed a novel mechanism of SREBP-1c-mediated cell cycle regulation, this study raises a number of important questions that require further investigation [8]. These experiments show that RNF20, a negative regulator of SREBP-1c, is down-regulated in ccRCC. However, it is still unknown how RNF20 expression is down-regulated in ccRCC. Recently, it has been demonstrated that epigenetic regulation of certain genes is important during the tumorigenic [26,27] and metabolic processes [28]. For example, the RNF20 promoter contains prominent CpG islands and is hypermethylated in human breast cancer [6]. In addition, miRNAs have been shown to control the expression of tumor suppressors and oncogenes in various cancers [29]. Thus, it seems that RNF20 expression might be reduced by epigenetic modifications or miRNAs in ccRCC. On the other hand, this work highlights the tumor-suppressive role of RNF20 in ccRCC by inhibiting SREBP-1c. To date, the requirement of the SREBP-1c for tumorigenesis has been reported in several solid tumors, including glioblastoma, breast, prostate, and colon cancer cells [4]. Inflammation is a key driver of cancer progression and also plays an important role in cancer initiation and promotion [30–36]. *RNF20* heterozygous null mice are prone to inflammation-mediated colon cancer [37]. According to these studies, it is possible that RNF20 suppresses tumorigenesis by modulating SREBP-1c in a number of solid tumors. Conversely, RNF20 promotes oncogenic transcriptional program in leukemia in which SREBP and fatty acid biosynthesis are suppressed [38,39]. Thus, future studies are needed to reveal whether RNF20–SREBP-1c axis would play central roles in various cancers. Clinically, ccRCC is resistant to cytotoxic agents widely used for cancer treatment. Instead, targetted therapies such as tyrosine kinase inhibitors, mTOR inhibitors, and immunotherapy are preferred for ccRCC treatment [40]. To overcome the limitations of conventional therapeutic approaches against ccRCC, many researchers have been studying the underlying mechanisms of ccRCC tumorigenesis. From a mechanistic standpoint, this is a very important finding what Lee et al. have elucidated in *VHL*-independent ccRCC tumorigenesis [8]. Therefore, it is likely that targetting RNF20–SREBP-1c pathway opens the door to another strategy against ccRCC therapy.

## Abbreviations

ACC: acetyl-CoA carboxylase
ccRCC: clear cell renal cell carcinoma
Cdk1: Cyclin-dependent kinase 1
CpG: 5′—C—phosphate—G—3′
ER: endoplasmic reticulum
FASN: fatty acid synthase
FLCN: folliculin
HIF: hypoxia-inducible factor
HMG-CoA reductase: 3-hydroxy-3-methyl-glutaryl-coenzyme A reductase
LDL: low density lipoprotein
mTOR: mechanistic target of rapamycin (mTOR)
PKA: protein Kinase A
PLIN2: Perilipin 2
Plk1: Polo like kinase 1
PTTG1: pituitary tumor transforming gene 1
RNF20: ring finger protein 20
SREBP-1c: sterol regulatory element-binding protein 1c
SCD1: stearoyl-CoA desaturase-1
TOFA: 5-(Tetradecyloxy)-2-furoic acid
VHL: von Hippel–Lindau
